# Spectral Properties of Foams and Emulsions

**DOI:** 10.3390/molecules26247704

**Published:** 2021-12-20

**Authors:** Andra Dinache, Mihail-Lucian Pascu, Adriana Smarandache

**Affiliations:** 1 National Institute for Laser, Plasma and Radiation Physics, 077125 Magurele, Ilfov, Romania; andra.dinache@inflpr.ro (A.D.); mihai.pascu@inflpr.ro (M.-L.P.); 2 Faculty of Physics, University of Bucharest, 077125 Magurele, Ilfov, Romania

**Keywords:** emulsion, foam, FTIR spectroscopy, Raman spectroscopy, surfactant, UV/Vis spectroscopy, DWS

## Abstract

The optical and spectral properties of foams and emulsions provide information about their micro-/nanostructures, chemical and time stability and molecular data of their components. Foams and emulsions are collections of different kinds of bubbles or drops with particular properties. A summary of various surfactant and emulsifier types is performed here, as well as an overview of methods for producing foams and emulsions. Absorption, reflectance, and vibrational spectroscopy (Fourier Transform Infrared spectroscopy-FTIR, Raman spectroscopy) studies are detailed in connection with the spectral characterization techniques of colloidal systems. Diffusing Wave Spectroscopy (DWS) data for foams and emulsions are likewise introduced. The utility of spectroscopic approaches has grown as processing power and analysis capabilities have improved. In addition, lasers offer advantages due to the specific properties of the emitted beams which allow focusing on very small volumes and enable accurate, fast, and high spatial resolution sample characterization. Emulsions and foams provide exceptional sensitive bases for measuring low concentrations of molecules down to the level of traces using spectroscopy techniques, thus opening new horizons in microfluidics.

## 1. Introduction

Foams and emulsions, part of the colloid class, have particular structures and contain fluids (either gases or liquids) or solid-state particles.

Liquid foams are metastable colloidal systems that appear as dispersions of gas bubbles in a continuous liquid phase. There is currently a lack of a broad theory to explain foam behavior. This is because the production of foam and its sustained existence involves a wide range of processes at various length scales, with interfacial phenomena playing a key role. Two interrelated terms are used when we refer to foam properties: foamability and foam stability. The ability of a surface-active agent solution to produce foams is referred to as foamability, whereas the foam stability represents the lifetime of a foam column [1].

An emulsion includes at least two immiscible liquids chosen so that one of them is dispersed as small droplets in the other. The diameters of the droplets are distributed usually between 100 nm (sometimes even smaller, down to some nm) and 100 µm. Their contents include specific molecular compounds and structures which may be associated with other chemicals that act as surfactants with different molecular composition with respect to the basic components [2,3]. The surfactants are used to stabilize the emulsions’ structures in view of applications, i.e., to keep the droplets immersed in the other liquid, stable and free of significant coalescence.

Emulsions are used in a large, ever larger, area of application in basic science and technological innovation where their optical and spectral properties, once measured, give valuable information about their molecular content and behavior at interaction with other structures/targets.

Among the applications of emulsions, one may mention food processing and production, petroleum and detergents technologies related to specific industries [4,5] and biomedicine [6]. In science, emulsions are intensely studied to better understand coalescence processes, interaction with any kind of tissues from biomaterials to sheets of artificial or natural origin, or even the behavior of construction materials in different environment conditions [7].

The optical and spectral properties of emulsions are important for applications since they give information about their micro- and nanostructures, chemical stability, time stability and components’ molecular data. The measurement of spectral properties is appealing since emulsions’ structures produce, upon interaction, longer optical paths of the light beams in emulsion samples and consequently, in many cases, more intense optical signals. This is particularly the case in laser-induced fluorescence (LIF) studies where one may observe that a sample of “clear” fluid yields lower intensity signals compared to an emulsion sample with the same dimensions and containing molecules at the same concentration [8,9]. The longer optical paths are due to light scattering at the interface between droplets with the surrounding materials which is completed by successive reflections and refractions. In some cases, emission is produced by the interaction of the light beams with droplets’ materials, and in others, with the droplets’ environment materials. Both processes contribute to the increase in the intensity of the emitted signals [8,10,11]. These characteristics are useful, since they allow, for instance, to identify the producers of the spectral effects at lower concentrations, down to traces, based on their molecular signatures.

The same kind of effects are obtained for foams, which are defined as systems that contain spreading/dispersions of gas bubbles in a basic environmental liquid or even solid. In general, for liquid media, the gas quantity/volume included in the solution is an important parameter which has a significant effect on the optical and spectral properties of the foams. The molecules of the two foam components, gas bubbles and liquid, are active in producing the optical and spectral effects in foams, but in most cases the emissions originate from the environmental liquid which is excited by beams dispersed by the gas bubbles. In principle, one may also obtain spectral information about the components of the gas bubbles in the liquid.

As in the case of emulsions, foams may be of natural origin, such as sea water foam [12], frog nest foam [13], beer foam and champaign [14], or artificial origin (biomedical and pharmaceutical foams) [15]. Besides the basic components, they may include nanoparticle structures such as gold or other metals [16,17]. The lifetime of the foams depends in all cases of the coalescence of the gas bubbles within the liquid, as well as the external factors that may influence the coalescence processes, such as environmental pressure, temperature, humidity and mechanical stability (vibrations). The prolonging of a foam’s lifetime, may be achieved by introducing surfactants into the liquid phase, i.e., molecular components that migrate towards the gas bubble–liquid interface.

These surfactants have also spectral properties and if optically active, they may play roles in the optical and spectral data harvested from foams. Literature shows that the most important spectral data which may be obtained from foams are in the field of optical absorption in the visible and NIR, but also in other spectral ranges, optically induced fluorescence and, in particular, laser-induced fluorescence and Raman spectroscopy. Reflectance spectra are also reported in some, cases [18].

This paper is a synthesis of results reported, mainly in recent years, about the above-mentioned spectral properties of foams and emulsions as well as their related phenomena.

## 2. Foams

Foam formation is a highly hydrodynamic process that necessitates the presence of surface-active agents which can adsorb at foam interfaces, lowering their free energy and, as a result, decreasing the overall free energy of such an interface-dominated system. Immiscible fluids (like liquids and gases, considered as such since gases in general may be dissolved in liquids in given proportions) can be formulated into a product only by stabilizing the interface surrounding the dispersed bubbles against coalescing or fusing [19]. Foams’ stability is therefore a critical subject in a variety of applications in environment and meteorology, foods, geology, agriculture, materials science, biology, medicine and pharmacy, petroleum production, mineral processing and home and personal care products [1,20].

The collapse of the foam is associated with three major destabilization mechanisms: (i) liquid drainage through thin films separating gas bubbles, mainly due to gravity and/or capillarity forces, resulting in thinner films; (ii) bubble coarsening (or Ostwald ripening) resulting from gas diffusion from smaller bubbles to larger ones, causing growth of the larger bubbles and a decrease in the overall number of bubbles; (iii) bubble coalescence occurring due to rupturing of thin films caused by insufficient elasticity, leading to a decrease in the number of bubbles and increase in their volumes [21].

The lifetime control of liquid foams, which presents significant interest in various research fields, including physical chemistry, materials chemistry, colloid science, nanotechnology, biochemistry, or medical applications is possible by adjusting the rate at which the three main mechanisms of foam destabilization work. Adjusting the foam lasting can be made by several methods, like changing solution conditions (pH, temperature, and ionic strength), using surfactants or application of an external field (light, magnetic and/or electric) [22].

From molecular point of view, the surfactant’s characteristics like chemical repeating unit, end functional groups, molecular weight and molecular weight distribution have distinct effects on a foam’s parameters [23].

Solubility is critical in many surfactant systems, especially for a homogenous series of straight chain aliphatic surfactants. Surface activity increases with chain length in the short alkyl chain length regimes, but above a critical value, solubility decreases with increasing chain lengths, resulting in a maximum or optimum value in surface activity arising from a balance between the two opposing effects. This is known as the Ferguson effect, a theory that sustains that a balance between lyophilic and lyophobic nature maximizes surface activity [24]. It was used to explain why an increase in the molecular weight of the linear alkyl chain of a homogeneous series of surfactants causes an increase in surface activity (foaming) until a decrease occurs at a critical chain length. This fact is important, not only for foaming, but also in processes such as detergency and emulsification [25].

### 2.1. Surfactants

Surfactants are important “molecular ingredients” used in foams. They may have significant influence on optical and spectral properties associated with microfluidic behavior (such as stability) and we will shortly present some of the most common compound classes used in this respect.

**Figure 1:**
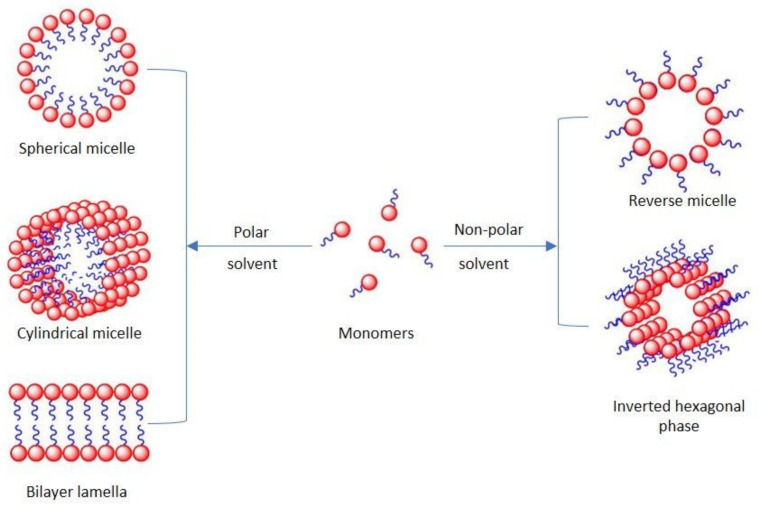
Surfactant molecular aggregates.

The surface-active agents are usually low molecular weight surfactants [26,27], but they can also be amphiphilic polymers [28], proteins [29], as well as their mixtures [30,31,32,33]. Their main role is to reduce the surface energy of the phase boundary. To be efficient, the foam stabilizer has to produce an irreversibly adsorbed elastic layer at that interface preventing film breaking between bubbles (coalescence), gas diffusion (coarsening), and gravity driven liquid flow (drainage) [34]. Surfactants have a very long history, the first records dating back almost three millennia BC [35]. They have even been the subject of investigation into the origins of life; meteorites containing lipid-like compounds have been found to assemble into boundary membranes and may be an interstellar prebiotic earth source of cell-membrane material [36]. Surface active agents are classified as amphiphilic compounds due to the presence of both hydrophilic and hydrophobic groups in their chemical structure [37]. The dual nature of the surfactants controls their assembly in the bulk. As shown in Figure 1, surfactant molecules can form aggregates including micelles, in which the hydrophobic tails compose the core of the aggregates and the hydrophilic headgroups are in contact with the aqueous phase.

Various types of aggregates including spherical or cylindrical micelles and bilayers can be found according to the spontaneous curvature of the surfactant monolayer [38]. Apart from micelles, surfactant molecules can also form other types of organized assemblies in solutions, for example, reverse micelles [39].

Low molecular mass surfactants are small molecules (with hydrodynamic radii of approx. 0.5–2 nm) containing a hydrophilic and a hydrophobic part. Typically, they are differentiated based on the polar group of the hydrophilic part. This part can be non-ionic [40,41,42] (uncharged) ionic [27] (cationic—positively charged, and anionic—negatively charged) or amphoteric [43] (zwitterionic—both positively and negatively charged). The charges of the amphoteric surfactants can be permanent or can be influenced by the pH of the medium to which they are exposed [44,45].

The effect of some non-ionic surfactants on the stability of polidocanol (POL) foams used in venous sclerotherapy (for instance) revealed that glycerin concentrations of up to 10% *v*/*v* and Tween80 concentrations of up to 20% could be of interest in terms of POL foam stability and its use in such medical applications [46].

Polymeric surfactants have far higher structural complexity than low-molecular-weight surfactants, which can lead to substantially different behavior of foams. For example, the number and distribution of hydrophilic and hydrophobic moieties along the chain may influence the polymeric agent’s surface activity [47]. Most of the polymeric surfactants reported in the literature are synthetic because it is very difficult to isolate this kind of compound from natural sources. However, proteins, which act as foams/emulsions stabilizers in natural systems are the most well-known examples of natural surfactants. Among them, caseins are a fast-developing family of natively unstructured proteins [48].

Recently, new surfactant molecules have emerged, and there is still room for novel compounds built for specific purposes and applications (such as nanoparticle synthesis and more diverse and environmentally friendly consumer products). The kind and positioning of extra functional groups are crucial for new functionalized surfactants. Slight changes in the molecular structure of traditional surfactants result in a rich morphology of foams that are investigated using increasingly advanced techniques, hence improving our understanding of their capabilities at the molecular level.

Surfactants are widely distributed in the environment. As organic pollutants, their toxicities have drawn extensive attention. The effects of anionic (sodium dodecyl sulphate (SDS)), cationic (dodecyl dimethyl benzyl ammonium chloride (1227)) and non-ionic (fatty alcohol polyoxyethylene ether (AEO)) surfactants on zebrafish larval behavior were evaluated by Wang et al. [49]. Their results revealed that 1227 and AEO at 1 μg/mL were toxic to larval locomotor activity and that SDS had no significant effects. All three surfactants incurred concentration-dependent response.

The skin toxicity of four ionic surfactants and fourteen non-ionic surfactants was investigated by Lémery et al. in connection to their structure/toxicity relationship. There was a clear difference between ionic and non-ionic surfactants. Ionic surfactants are the most toxic if they are soluble in water. Crystalline ionic surfactants of low solubility show low toxicity. Since the molecular parameters of ionic, non-ionic, water-soluble and crystalline surfactants are different, a universal parameter was introduced, the order parameter, describing the orientation ordering of surfactant molecules at interfaces [50].

### 2.2. Particles as Emulsion and Foam Stabilizers

The study of nanometric particles and their interaction with fluid interfaces is an interesting and topical research subject in the field of their applicability in colloids domain [51,52,53]. Nanoparticles (NPs) are employed frequently in association with surfactants, as stabilizing agents of disperse systems like foams and emulsions [54,55]. Many experimental and theoretical papers are available in the literature about the nanostructure of foam systems, however the basic mechanisms underlying the stabilizing effect of NPs is still a topical issue [56].

The use of NPs may offer an alternative to surfactants used for foam and emulsion stabilization, especially in the presence of oil. The NPs can strongly adsorb at the interface and stabilize foams at high temperature and salinity [53,57,58]. A new generation of NPs has been manufactured using affordable and low-cost raw materials such as fly ash or silica [59]. The critical parameter for SiO_2_ NPs in the elaboration and stabilization of liquid foams is their hydrophilic or hydrophobic character (property related to wettability) and the three-phase contact angle (measured with respect to water). It was found that the maximum diameter of particles able to stabilize liquid foams is below 3 µm [60].

Shojaei et al. have investigated the effects of surfactants with different charges (anionic, cationic and non-ionic) on foam stability in the presence of charge-stabilized silica (SiO_2_) NPs. Their results show that the nature and magnitude of the stabilization strongly depend on the nature of the surfactant, its concentration and the concentration of NPs. Both results from the bubble-scale and the bulk-scale experiments suggest that compatibility tests between surfactants and NPs are a pre-requisite to obtain stable foams [61].

The synergistic effect of a surfactant and NPs or the modification of the surface of solid NPs through physicochemical interactions with surfactants may enhance foam stability and generate stronger foams than the use of surfactants alone. Several studies reported the ability of mixtures of surfactant and NPs to enhance foam stability [62,63].

A promising drug delivery approach to deal with conventional cancer therapy drawbacks includes the application of multifunctional nanotechnology-driven drug delivery systems, where micelles, drug conjugates, NPs and nanomaterials have shown important advances. In this regard, the development of a novel nanoscale drug delivery system-based nanotherapeutic that combines chemotherapy and photodynamic therapy using 660 nm light irradiation into one single platform to achieve synergistic anticancer properties to overcome cisplatin resistance was reported. Mesoporous silica NPs (MSNs) with diameters of about 100 nm and slightly positive surface charge were used as drug delivery vector to conjugate cisplatin prodrug and to load the photosensitizer chlorin e6 (Ce6) to enable a dual drug-loaded delivery system MSNs/Ce6/Pt [64,65]. Kumar et al. report the development of a 100 nm MSNs-based enzyme-responsive material for colon-specific drug delivery. Guar gum, a natural carbohydrate polymer was used as a cover layer to contain a model drug, such as 5-flurouracil (5FU) within the mesoporous channels of MSN. It was shown that MSNs maintained their discrete nanoparticle identity after guar gum capping through non-covalent interaction. The release of 5FU from guar gum capped MSN was specifically triggered via enzymatic biodegradation of guar gum by colonic enzymes in the simulated colonic microenvironment [65].

Surfactants have an impact on the physicochemical characteristics of NPs that goes beyond stability. Surface phenomena induced by surfactants have a significant impact on their interactions at the cellular level [66]. As a result, depending on the type of surfactant, the interaction with cells can be increased or decreased. Voigt et al. conducted a blood–retina barrier passage study as a blood–brain barrier (BBB) model of fluorescent polybutylcyanoacrylate NPs with different types of surfactants (non-ionic, anionic and cationic), size (67–464 nm) and zeta-potential. NPs’ size and charge had no influence on BBB passage and cell labelling. Moreover, in the context of NPs with reduced size (down to 87 nm) no BBB crossing was observed, even adding SDS to the non-ionic surfactant [67].

### 2.3. Methods of Foam Generation

During foam formation, the surface energy *E = σA* increases with the appearance of gas–liquid interfaces having the surface tension, σ, and interfacial area, A. The increased surface energy means that the foam formation is not a spontaneous process, and the input of energy is indispensable to generate a column of foam. The resulting network of gas/liquid interfaces provides liquid foams with numerous complex and useful properties. Amongst these, strong scattering of light is one of the most important foam features [68].

Different foaming techniques are known depending on the procedure by which energy is introduced into the system. They make use of the fundamental mechanisms of bubble generation described in detail by Drenckhan and Saint-Jalmes [69]. The type of foam that is produced is influenced by the generation procedures that use parameters such as process velocity, gas intake, turbulence, temperature, liquid viscosity, surfactant type and rate of surfactant diffusion to the interface. All these have an impact on bubble size, stability, and foam microstructure. The best-known foaming techniques can be classified according to physical, chemical or biological processes used to generate the foam [23].

The physical foaming techniques are the most used, and consist in mechanical foaming (bubbling, foam generation in porous media, wave breaking, shaking, rotor–stator mixers, and double syringe techniques), and phase transition (cavitation, nucleation).

The foam generation through the simplest traditional mechanical methods conducts to large bubbles with wide size distributions (ranging from 0.1 to 3 mm). In these processes, admission of gas occurs from the surface during the incipient agitation. Large bubbles are produced, which are broken down by continuous shearing forces until equilibrium is established [70]. The difficulties in producing small bubbles can be explained by the fact that deformation of a large bubble to produce smaller bubbles is opposed by an increase in Laplace pressure. To carry out an effective disruption process, a high external stress (equivalent or exceeding the Laplace pressure) needs to be supplied by a velocity gradient [25]. To ensure a successful bubble generation step, a sufficient amount of surfactant must be supplied to the gas–liquid interface to create a surface tension gradient (high Gibbs elasticity) that acts to stabilize the fresh bubbles. [71].

Smaller quantities of foams, but with a very precise control over the gas fraction, can be obtained using the double-syringe technique, that are mainly intended for clinical use. One of the methods to generate microfoam for veins sclerotherapy is the Tessari technique, which uses a double syringe system bounded through a three-way connector. The two-phase mixture is pushed back and forth through the double-syringe system. At the outset, one syringe contains the gas, while the other one contains the foaming liquid. Both mix efficiently after a few cycles, giving rise to foams with very small bubbles (around 10 μm) and with well-controlled gas fractions [72].

Also, part of physical foaming techniques, cavitation, or high-intensity agitation is one of the widely used methods of generating nano-bubbles. There are several different methods of producing this process: (a) hydrodynamic cavitation by fluid flow, (b) acoustic cavitation by acoustic energy, (c) optical cavitation by using photons from a laser beam and (d) particle cavitation, induced by elementary particles. In this context, the study of Wu et al. demonstrated that the baffled high-intensity agitation device they designed is able to generate submicron size (less than 500 nm) bubbles through hydrodynamic cavitation. This process is influenced by factors such as agitation speed and time, temperature of solutions, dissolved gas content and water chemistry. The presence of surfactant improves the process, increasing both the number of bubbles and the stability of foam [73].

Laser-induced cavitation is an effective technique to generate controlled cavitation bubbles, both for basic studies and for applications. Cavitation in liquids is an interesting phenomenon with substantial impact in engineering, medicine and fundamental physics [74]. Except the association with erosion damage in hydraulic turbomachinery [75], foams generated through cavitation find interesting microfluidics [76], as well as biomedical applications like cell poration [77] and drug delivery [78].

**Figure 2:**
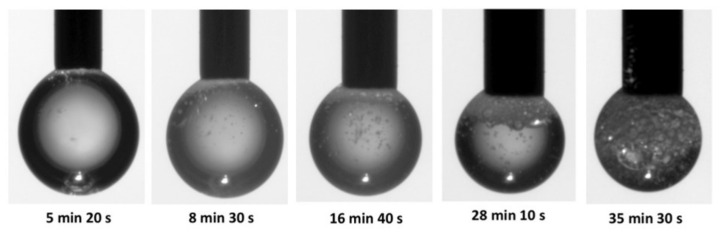
Foam generation in aqueous Vancomycin droplets (V_d_ = 5μL) during the second harmonic (λ = 532 nm) of Nd:YAG fundamental laser irradiation.

For a vancomycin solution exposed to laser radiation in bulk as well as in droplet presentation, a structural modification was observed, that is accompanied by foam generation (Figure 2). Out of the experimental conditions, the beam energy and the laser pulse time width are parameters with the most important impact on foam generation. The minimum value of beam energy for which foam was obtained is 40 mJ for a 0.5 mL and 7 mJ for 5 μL. The obtained foam was stable for several minutes after stopping laser exposure [79]. The mechanism involved in foam generation may be the laser ablation in liquids. Studies have shown that short pulses lead to photomechanical ablation, together with cavitation beneath the surface due to a transient thermoelastic pressure, while for pulses longer than the acoustic relaxation time, energy accumulation leads to an increase in local temperature that produces smaller droplets that are afterwards expelled from the solution [80]. In Figure 2, droplets containing vancomycin foams are shown, when the solution is exposed to laser radiation for time intervals up to tens of minutes. The initial vancomycin solutions in water are generated as microliter droplets in pendant positions suspended by a capillary which afterwards are exposed to a pulsed green laser beam.

Bubbles are generated into the vancomycin solution due to the acoustic wave that accompanies the laser pulse that produces nucleation into the liquid. No important temperature variation was registered during the experiment, so the “cold ablation” foaming process was considered to be responsible for the vancomycin behavior observed at interaction with laser radiation [6].

Chemical reactions such as electrolysis are an alternative to generate small size bubbles. In this case, bubble generation is largely dependent on the ionic strength and temperature of solutions [81,82]. The limited range of gases that can be produced is a significant impediment for applications of bubble and foam generation by electrolysis. It is usually oxygen or hydrogen, which severely limits their application possibilities. Alkaline water electrolysis using porous foam metals as electrodes was performed when a magnetic field was applied [83]. It was found that the energy consumption of water electrolysis was considerably reduced at high current density by magnetohydrodynamic convection. One of the main applications of this foaming technique is electro-flotation whose efficiency increases with decreasing bubble size since the total interfacial area is inversely proportional to the bubble size. Wastewater treatment or mineral flotation at small scales are the principal domains in which this method is used [84].

The most common biological foaming approach relies on gas-generating species such as yeast that may serve as model to study CO_2_ behavior under pressurized conditions with impact on fermentation biotechnology [85]. Yeast is involved in wine and beer foaming processes when the secondary fermentation takes place over a long period of time, usually for several months [86]. The released compounds include amino acids, peptides, proteins, and polysaccharides, all of which are known to be involved in foam formation and stabilization. Among the released proteins, mannoproteins (glycoproteins located in the outermost layer of the yeast cell wall, where they are connected to a matrix of amorphous *β*-1,3-glucan by covalent bonds) derived from the yeast cell wall, are particularly important as their hydrophobic nature causes them to preferentially adsorb to the gas/liquid interface of foam bubbles [87,88]. Ethanol and CO_2_ are produced when yeast ferments wine must or beer wort. Both affect foam production in different ways: more ethanol produces less foam. On the contrary, the more CO_2_ dissolved, the more foam generated. This is because yeast cell walls adhere to the gas bubbles, resulting in a foam layer at the fermentation tank’s surface [89].

Responsive liquid foams and bubbles refer to systems for which the stability, structure, shape and movement can be controlled by the application of stimuli (pH, temperature or ionic strength) which can change molecular behavior of solution components, or by the application of an external field (light, magnetic or electric field exposure) [90]. Surfactants, peptides, polymers, soft polymer particles, surfactant self-assembly, crystalline particles, emulsion droplets and solid particles have all been described as foam stabilizers used to design these responsive foam systems [22].

### 2.4. Spectral Studies of Foams

Despite the importance of understanding foam structure at all length scales, only limited experimental techniques have been used in its study. Simulation studies focused on understanding the mechanics of foams under shear stress [91] have revealed the necessity for more precise experimental procedures to collect essential information on foam structure.

The optical processes, like absorption and scattering, jointly govern the light propagation in turbid environments. In this respect, the study of optical properties of surface-active agents might be useful in order to elucidate the mechanisms involved in foam generation and its behavior in connection with different external parameters that may affect foam characteristics. To further understand their function in foam formation, Xiang and al. [92] investigated the release of non-cellulosic components (cell wall heteropolysaccharides, lignin, and extractives) from swollen wood fibers in the presence of an anionic surfactant (SDS) at submicellar concentrations. Between SDS and the leached, non-cellulosic components, highly surface-active aggregates develop, which do not form in the presence of cationic or nonionic surfactants. Using analytical techniques at the interface as well as optical approaches such as UV-Vis Spectroscopy and Attenuated Total Reflection Fourier Transform Infrared Spectroscopy (ATR-FTIR), the in situ and efficient formation of liquid foams in the presence of the leached species was proven. By comparing the respective ATR-FTIR spectra with those obtained from SDS and referenced hemicelluloses [93], the characteristic peaks of hemicellulose are identified at 3355 (O–H bond stretching), 1040 (C–O bond stretching of the ether groups), and 897 cm^−1^ (*β*-1,4 glycosidic bond stretching). Additionally, the peak at 1215 cm^−1^ was assigned to the stretching of skeletal vibration of S–O in SDS. The presence of lignin in hemicellulose was confirmed by the UV-Vis absorbance analysis with a maximum intensity at 205 nm. The foaming capacity, foam stability and structure were all determined as a function of the aqueous suspension’s composition. The results suggest that only aqueous solutions of the anionic surfactant can remove naturally occurring components attached to wood fibers. They can also generate high-foaming surface-active aggregates [92].

In an attempt to develop multifunctional microwave absorbers that worked in complex environments, He et al. proposed a hybrid foam with a high light adsorption capacity that is promising for use in photo-thermal conversion or as a photoresponsive material. The photo-thermal conversion properties of the hybrid foam were investigated based on the transmittance and diffuse reflectance spectra [94].

The effects of different surfactants (polyvinyl alcohol-PVOH, SDS, cetyl-trimethyl ammonium bromide-CTAB) and gases (N_2_ and CO_2_) on the ability of foams to coalesce and remain stable in the context of their applications in the pulp and paper industry was studied using high-speed camera observations and FTIR spectroscopy. Based on the FTIR spectra analysis, the results showed that when the liquid film was newly formed, the corresponding peak of O–H group vibration at 3400 cm^−1^ for SDS was the strongest, followed in order by those for CTAB and PVOH. The evolution in time of FTIR spectra indicate a quick liquid drainage process for both samples based on SDS and CTAB surfactants while the absorption peak of O–H decreased slowly when PVOH was used, whic indicates good stability foam in this case. These results are further linked with the gas diffusion rate of the foam with impact on its stability [95].

The stability of decontamination foams that contain a chemical reagent is a necessity for their usage in nuclear power plant decontamination. The effects of adding silica NPs modified with various functional groups, such as propyl (–CH_3_), amine (–NH_2_) and thiol (–SH) on decontamination foam stability was recently reported [96]. The surface properties of these silica NPs were characterized using ATR-FTIR analyses. Because of their extensive dispersion in the liquid layer, the amine-modified silica NPs’ agglomeration in such foams is weaker than that of the other modified silica NPs. Furthermore, at pH = 2, the foam containing amine-modified silica NPs was shown to be stable for 60 min, indicating that it might be used for decontamination. The study found that the decontamination foam with amine-modified silica NPs has the best foam structure of the three investigated foams. The foam stability is improved by the well-dispersed and smaller amine-modified silica NPs, which act as a barrier between the gas bubbles and prevent their coalescence. The thiol and propyl-modified silica NPs create large-diameter aggregates that diminish the maximal capillary pressure of coalescence and hence reduce foam stability [96].

It has been reported that polyurethane foams functionalized with sulfonic acid groups have been utilized to remove lead (Pb^2+^) ions from aqueous solutions [97]. The functionalized polyurethane foam characterized by FTIR spectroscopy indicated that the sulfonic acid functional groups were successfully integrated into the polyurethane backbone.

Sclerosant medicines that have been foamed are frequently used in biological applications [98,99,100,101,102]. The most popular medicines used worldwide for varicose vein foam sclerotherapy are detergent-like sclerosants. By displacing intravascular blood, the injection of foamed medications maximizes the interaction of the active substance with the vessel wall. As a result, the medicine is diluted and deactivated as little as possible by blood components. In addition, a lower concentration of active ingredient is required for therapy. Results regarding the elements that may influence the foam stability of the sclerosing agent are discussed in [103] with the aim to better understand the physical processes involved in the evolution of foaming polidocanol (POL) for further biomedical applications. Foam stability improves with an enhancement of sclerosant concentration and an increase in air percentage.

**Figure 3:**
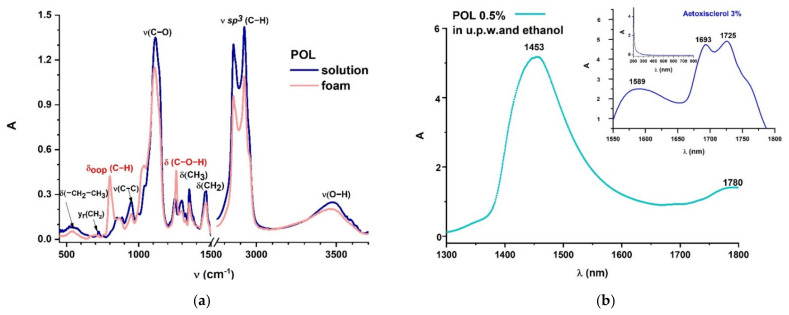
Optical characterization of POL: FTIR spectra of both solution and foam sample showing the vibrational changes of molecules induced by foam generation procedure (Tessari’s double syringe method) (**a**), and UV-Vis-NIR absorption spectra, with the spectrum of commercially available Aetoxisclerol (Kreussler Pharma) (**b**).

In both fresh and laser irradiated samples, the FTIR spectra reveal molecular structural changes of foam when compared to liquid POL. It is interesting to observe that when POL is foamed, C–H out-of-plane bending vibrations occur, and C–O–H bending vibrations are also influenced (Figure 3a). The optical characterization of POL based on the absorption spectrum registered in UV/Vis/NIR spectral ranges highlights absorption peaks centered in NIR, while it totally transmits in UV/Vis. Previous research has yielded similar results for commercially available Aetoxisclerol (Kreussler Pharma), which contains POL as an active ingredient, owing to the superimposed absorption properties of all the compounds present in the drug solution (Figure 3b) [104].

Raman spectroscopy is a powerful noninvasive technique to assess the structure and dynamics of a system at molecular level. Despite its powerful characterization capabilities, Raman spectroscopy has not been widely used in the study of foams. Because surfactant molecules are so important in the formation and stabilization of foam, Raman spectroscopy, with its ability to monitor molecular vibrations, can provide critical information on their packing, mobility, and conformation [105].

Raman scattering is caused by deformation/stretching of different vibrational bonds of molecules. If the macroscopic and microscopic properties of a foam are linked, the analysis of Raman line profiles is able to indirectly assess its elastic properties by investigating its molecular inner activity.

The work of Zhao et al. presents the graphene foam characteristics obtained by using Raman spectroscopy [106]. The Raman spectrum of 3D graphene foam displays two characteristic peaks: at 1583 cm^−1^ (G band), due to the doubly degenerate zone center E_2g_ mode, and 2710 cm^−1^ (2D band). No obvious graphene D band at ~1350 cm^−1^ was observed, thus indicating that the graphene foam is of high quality. The D band is used for the characterization of defects or the disorder of the graphene, its density being proportional to the amount of disorder in the sample [107].

Different studies on wet foam have explained the gross properties of wet foam in the light of its characteristic molecular structure using Raman measurements. Their results highlighted the observed shift in the low frequency Raman peak position of the methylene rocking mode with the variation in internal stress in the foam. Wet foam exhibits a steady structural shift from an all-trans conformation to a crystalline structure as it ages, according to Raman measurements in the 1000 cm^−1^ to 1450 cm^−1^ region [108]. Drainage of water from wet foam is discussed and, in addition to free water molecules which drain out with ageing of foam, water clusters of only a few water molecules are also present in foam. The correlation between the internal stress and the characteristics of a vibrational mode in wet foam is also shown. The fundamental challenge in utilizing Raman spectroscopy to examine wet foam is due to multiple light scattering within the bubbles. This affects the Raman signal from the bubbly structure, impacting the signal to noise ratio in the spectrum. To overcome this difficulty and provide an optimum Raman signal collection, the spectrometer slit width should be adjusted to match the mean bubble size of foam [109]. The investigations carried out by Goutev and Nickolov demonstrate the capabilities of Raman spectroscopy to study the microstructure of three-dimensional foams and their dynamics. The Raman experiment set-ups were performed in backscattering, the laser beam making an angle of 50° with the cell. The foam was placed in a closed glass rectangular cell to prevent changes due to evaporation or was probed directly in the case of dry or semidry residue. The liquid that drained off the foam was studied in 2 mm internal diameter capillaries in the conventional 90° geometry with the laser beam parallel to the long axis of the capillaries. The scattered signal was collected by a lens (F = 40 cm), passed through a Raman notch filter and imaged on the entrance slit of a single 60 cm focal length polychromator. Detection of spectra was accomplished by an intensified vidicon and computer controlled multichannel system. An argon ion laser emitting at 488 nm was used as excitation source. The beam with typically 120 mW was focused on the sample using a lens (f = 100 mm). The detection system was calibrated by Ne emission lines. It is seen from the Raman spectra of ‘‘wet’’ foam in the C–H region (2800–3000 cm^−1^) that the band shape changes weakly with aging. Its subcomponents are comparatively well expressed in all stages of foam evolution and only their relative intensities change [109].

Aetoxisclerol solution and foam samples were analyzed by Raman spectroscopy in an attempt to improve the efficacy of the laser sclerotherapy for small varicose veins if the sclerosing agent is used as foam. The Raman vibrational lines associated with foam samples are more organized and powerful than those associated with liquid specimens [68]. When a laser beam interacts with a 3D foam, the movement of light through this scattering medium is essentially a random process with a mean free path, l*, which is referred to as diffusive propagation. This causes so-called diffusive excitation, which results in a distribution of elementary Raman scattering centers in the bulk of the foam. In turn, the Raman signal will diffuse in all directions, reaching in the end the foam cell boundaries and allowing the Raman signal from the bulk foam to be detected. According to the relationship l* = 3.5 × *d* [110], the specific dimension of the Raman intensity distribution at the scattering focal plane (usually the surface of the foam) is proportional to the transport mean free path l*, which has been linked with the size of the foam bubble (*d*) [111].

**Figure 4:**
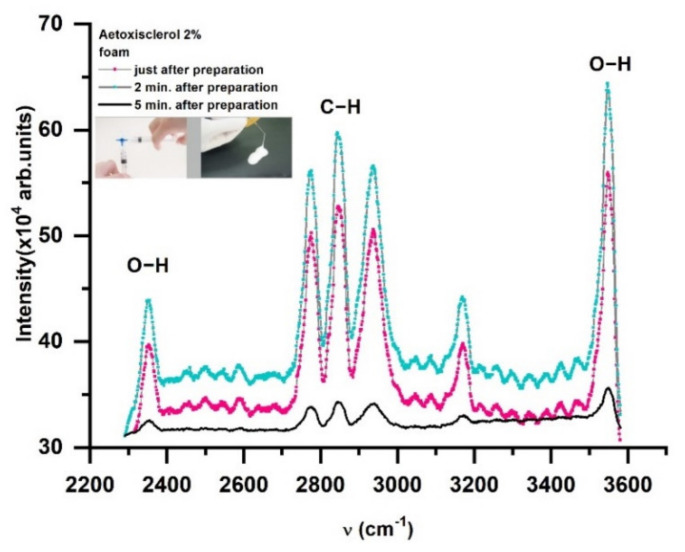
The influence of foam stability on the Raman spectra of Aetoxisclerol sclerosant drug.

Raman spectra were also recorded at various times after foam samples were prepared. The Raman spectra (Figure 4) appears to shift very quickly, with Raman lines 10 times less strong than initial values after 5 min of foam generation.

The findings suggest that various aspects, such as bubble diameters related to foam cohesiveness, must be taken into account when considering the temporal moment of exposure of the varicose vein injected with foamed medication and exposed in the tissue to laser radiation [68].

The multiple scattering of light by aqueous foams was systematically studied as a function of wavelength, bubble size, and liquid fraction. Results are analyzed in terms of the transport mean free path of the photons and an extrapolation length ratio for the diffuse photon concentration field. An experimental study of the dependence of these parameters on structure and composition of foams was detailed in Ref. [112]. The transport mean free path was found to be proportional to the bubble diameter and the reciprocal of the square root of liquid fraction. The extrapolation length ratio varies almost linearly with liquid fraction between the values for water–glass–air and air–glass–air interfaces.

Diffusing Wave Spectroscopy (DWS) can be employed as an optical rheology tool with numerous applications for studying the structure, dynamics and linear viscoelastic properties of complex fluid systems like foams and emulsions. Improved DWS based on the automatized determination of the optical transport and absorption mean free path was reported in [113] by simply measuring the photon count rate of both the light scattered in transmission and backscattering geometry.

The gas–liquid or liquid–liquid interfaces substantially scatter light propagating in foams or emulsions. This property makes it difficult to directly detect the structure and dynamics deep within the bulk of such samples. Multiple light scattering, on the other hand, can be used to develop non-invasive experimental approaches for measuring average bubble size, droplet size, and dispersed volume fraction. When a laser is used to illuminate a sample, the transmitted or backscattered light generates a speckled interference pattern, revealing the dynamics of intrinsic structural changes (coarsening, flocculation or external stress) through temporal variations [114].

Diffuse Transmission Spectroscopy (DTS) was introduced by Kaplan et al. to investigate the structure of opaque colloidal suspensions [115]. This technique is suitable for determining the temporal dynamics of average bubble dimensions during foams’ coarsening or of the liquid fraction of a foam during drainage [116].

For investigation of foams, DWS gives information about stationary dynamics of bubbles reconfigurations in time, as an average of the whole sample through the transmitted light or just near the surface of the foam, through backscattered light [114].

Thus, DWS was employed to probe the reorganization of bubbles after an aqueous foam is subject to transient shear deformation, determining that the bubble dynamics returns to the behavior of a stationary foam via a nonlinear relaxation depending on the age of the foam and amplitude of shear [117]. In earlier studies, when shear stress was applied to shaving cream foam, DWS showed that the decay of the correlation functions is associated with intrinsic rearrangements of bubbles [118].

In [119] DWS was employed to investigate the elastic response of an aqueous foam (shaving cream) when it was subjected to oscillating shear strain. It was observed that for small amplitudes of the strain, the response in bubble rearrangement is linear, but if the strain amplitude is larger than 0.05% the response is nonlinear.

Also, DWS provided insights about the bubble dynamics when shear stress is applied continuously to an aqueous foam. It was discovered that the bubble dynamics depend on the relationship between the strain rates and the macroscopic deformation. For slow shear, the deformation is due only to localized rearrangements of bubbles occurring because of foam ageing and applied shear; for intermediate shear, the deformation appears because of bubble reorganization induced by nonaffine and directed shear; and for fast shear, the foam bubbles are moving constantly, leading to “melting” of the foam [120].

DWS analysis of the coarsening of an aqueous foam loaded with monodisperse latex beads revealed that the temporal autocorrelation function of the scattering intensity presents two decays decoupled and separated in time. These distinct decays are due to two different processes: bubble rearrangements during foam ageing, which is responsible for the longer decay, and Brownian motion of the colloidal particles in the liquid fraction, giving rise to the shorter decay [121].

Marze et al. showed that DWS helps distinguishing between foams subjected to slip and foams subjected to shear. The slip velocity was determined to be maximum at the yield stress [122].

Multispeckle DWS was employed to study the bubble dynamics during coarsening of levitated foams. Different liquid fractions were analyzed, showing that local bubble reorganization dominates the dynamics of dry foams, but the bubbles have a ballistic motion for high-liquid-fraction foams. Multispeckle DWS enabled studying the non-local dynamics at different times, showing that during ageing of dry foams, a substantial reorganization of bubbles is responsible for intermittent bursts observed in the bubble dynamics. Opposite to this, for wet foams, the large-scale dynamics of bubbles undergo ballistic and convection motions [123].

All these spectroscopy techniques proved to offer valuable information in foam analysis.

## 3. Emulsions

Emulsions are defined as dispersed systems, constituted of two immiscible liquids, one being the dispersed phase and the other the continuous phase. In other words, emulsions are colloids with both phases being liquids, usually stabilized by an emulsifier [124,125,126].

A classification of emulsions can be made taking into consideration several factors. First, regarding the dispersed and continuous phases, emulsions can be classified into three types: a) oil-in-water (O/W), also called water-based or direct emulsions; b) water-in-oil (W/O), also called oil-based or inverse emulsions; and c) multiple emulsions, which can be water-in-oil-in-water (W/O/W) or oil-in-water-in-oil (O/W/O) [127,128].

An important criterium for classification is the size of emulsions’ droplets. With respect to the size, there are three types of emulsions: macroemulsions (classical emulsions), nanoemulsions (miniemulsions), and microemulsions. For a period of time, in the case of self-emulsifying systems proposed for drug delivery many studies confused nanoemulsions with microemulsions [129]. The terms “nanoemulsions” and “microemulsions” might be misleading, considering the dimensions of their droplets, but this is due to the fact that “microemulsion” appeared first in the timeline of colloids scientific articles. The term “nanoemulsion” was introduced after approximately 35 years [125,130,131].

For a better differentiation, are summarized further the characteristics of each category.

Macroemulsions are thermodynamically unstable and weakly stable from the kinetical point of view. The droplets of the emulsions are spherical, they present high polydispersity (usually >40%), with sizes between 1 and 100 µm [128,132]. Nanoemulsions, similar to classical emulsions, are unstable in thermodynamic terms, but due to the very small droplet dimensions, their destabilization is so slow that one may say that nanoemulsions are kinetically stable. Spherical shaped droplets of nanoemulsions have sizes between 20 and 500 nm [133], although scientific reports propose different size intervals, e.g., 20–200 nm [134], 100 nm–1 µm [128], 10–300 nm [129], etc. Nanoemulsions can be found in colloid literature also as submicron emulsions, miniemulsions or ultrafine emulsions [134,135]. They have typically low polydispersity, usually <10–20% [132].

Microemulsions are stable from the thermodynamic point of view and have structures ranging between 10 and 100 nm in size, with a variety of shapes, e.g., spherical micelles or reverse micelles, cylindric worm-like rod-micelles, lamellar structures, bicontinuous sponge-like, hexagonal, etc [125,129]. The polydispersity of the microemulsions is very low, usually <10%, demonstrating a uniformity in the sizes of constituent structures [132]. Further, microemulsions are classified as follows: a) Winsor I—lower swollen micelles in water and upper excess oil phase (biphasic); b) Winsor II—lower excess water phase and upper W/O emulsion (biphasic); c) Winsor III—lower excess aqueous phase, middle bicontinous emulsion, upper excess oil phase (triphasic); and d) Winsor IV—microemulsion phase (monophasic) [129,136,137,138]. Microemulsions are formed spontaneously, and depending on the emulsifier, temperature and salinity, they can transit from one Winsor type to another [138].

### 3.1. Emulsifiers

Emulsifiers stabilize the emulsions by decreasing the interfacial tension and the strength of the interfacial film. Their role is to assemble at the interface between oil and water phases, forming a barrier that keeps the droplets from coalescing [124,139]. Emulsifying agents can be surfactants, proteins, lipids, polymers or solid particles. In the particular case when emulsions are stabilized only by solid particles, they are called Pickering emulsions [132,139,140].

Surface active agents are molecules that can self-assemble in micelles in a liquid and adsorb to the interface between oil and water phases, in the case of emulsions. In order to do that, surfactants have two functional groups, one hydrophobic, or lipophilic, that orients towards oil phase, and another hydrophilic, with affinity to water [141].

Ionic surfactants are typically hydrophilic, whereas the solubility of the non-ionic surfactants depends on hydrophilic–lipophilic balance (HLB) [141,142].

The solubility of the surfactant will influence the type of emulsion, in the case of almost equal volumes of water and oil phase. According to Bancroft’s rule, the continuous phase of an emulsion will be the one in which the surfactant is most soluble [142,143].

Non-ionic surfactants are preferred in formulating microemulsions due to their uncharged head groups and their better resistance to changes of pH or salinity. In addition, non-ionic surfactants are considered safer than the ionic ones for ingestion [138]. For a greater effect, a co-surfactant can be utilized in formulating emulsions [144].

In case of Pickering emulsions, the adsorption of solid particles at the surface of the droplets stabilizes the emulsions, preventing coalescence and Ostwald ripening. Solid particles employed in stabilization of Pickering emulsions include hydroxyapatite NPs, silica, clay, magnetic Fe_3_O_4_ NPs, carbon nanotubes and chitosan NPs [140,145]. Besides inorganic particles, biological particles such as derivatives of polysaccharides or proteins have also been used as emulsifiers [139,145]. The effect of surfactants on Pickering emulsions was presented in several studies. For example, low concentrations of Tween 80 and high concentrations of soybean lecithin enhanced the stability of cyclodextrin Pickering emulsions, but soybean lecithin at low concentrations inhibits the formulation of these emulsions [146]. Moreover, addition of Tween 20 to a Pickering emulsion stabilized by zein NPs improves the stability of the emulsion [147].

### 3.2. Techniques of Emulsification

There are mainly two categories of emulsification techniques: high-energy and low-energy emulsification methods. High-energy techniques include high pressure homogenization (HPH), microfluidization, high-energy stirring, ultrasonication, membrane emulsification and laser-assisted emulsification. In some cases, two methods are combined, e.g., stirring and high-pressure homogenization. The most used low-energy methods are emulsion inversion point (EIP) and phase inversion temperature (PIT) [132,148,149,150]. If a combination of two methods, high-energy and low-energy emulsification is employed, it is possible to generate reverse nanoemulsions [151].

High-energy emulsification methods achieve size reduction of emulsion droplets by employing disruptive forces. For the HPH method, first, an emulsion is generated with an ultraturrax and afterwards it is pushed through a very narrow gap (dimension < 10 µm), causing a decrease in the droplets’ size due to extreme elongation and shear stress. The advantage of this method is that it can be repeated an unlimited number of times [132,152,153].

Microfluidization method is similar to HPH. Nanoemulsions are generated with a microfluidizer, where macroemulsions are pumped through microchannels. The generated emulsion is finally filtered to remove larger droplets. This method can be also repeated several times, until droplets reach the desired dimensions [132,153,154].

Ultrasonication employs sound waves with frequencies higher than 20 kHz to rupture the droplets in a macroemulsion. This happens due to the pressure created by the shock waves produced in the implosion of the cavitation bubbles originating from the ultrasonic waves [132,152].

The membrane emulsification method involves drop-by-drop passing through a microporous membrane. This way, the dispersed phase is pushed through the pores of a membrane in the continuous phase, formulating an emulsion with droplets sizes determined by the size of the pores. Membrane emulsification can be divided in two categories: moving continuous phase and moving membrane [149,155].

**Figure 5:**
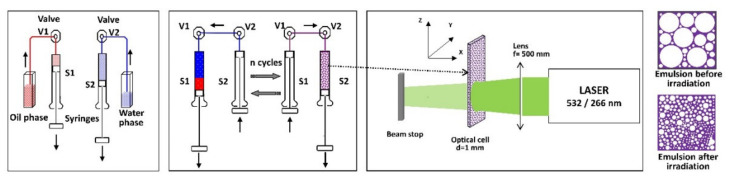
Principle of a new laser-assisted emulsification method. Step one: mixing of the continuous phase with the dispersed phase with a double syringe method. Step two: non-resonant interaction of the coarse emulsion with the laser radiation.

Laser-assisted emulsification was recently developed, and is a two-step method (Figure 5). First, the continuous phase and dispersed phase are mixed together in a double syringe system. This system was modified from a diluter-dispenser system and the software designed specially allows to control the emulsification process. The system allows the user to select the needed parameters for emulsification: loading speed for the continuous phase and dispersed phase, the volumes of the two phases, number of mixing cycles and the expelling speed of the emulsion. The obtained emulsion is then placed in a narrow cuvette (optical path of 1 mm) and exposed to laser radiation of a pulsed Nd:YAG laser at 10 pps and 6ns pulse duration. The employed average energy of the laser beam was 35 mJ and the exposure time was 1h. The beam wavelength was 532 nm, chosen so that the components of the emulsion do not absorb at this wavelength. This way, the effect of the laser radiation on the emulsions is strictly mechanical, leading to generation of emulsions with smaller sized droplets (around 150 nm in diameter) [150].

This method has the advantage of using low volumes of solutions, even hundreds of microliters, making it suitable for biomedical applications. In addition, mixing cycles, speeds, laser energy and irradiation time are customizable, allowing to obtain emulsions with micrometer and nanometer size droplets [150].

Opposed to high-energy methods, low-energy methods use the internal energy of the emulsion to generate smaller droplets. For example, the PIT method depends on the modification of non-ionic surfactants’ affinities for water/oil with variation of temperature [153,156].

### 3.3. Spectral Properties of Emulsions

As described previously for foams, spectroscopy techniques can provide useful information in characterization of emulsions, as well.

It is known that the UV-Vis absorption spectrum of an emulsion can give information about the absorption and scattering properties of the droplets. UV-Vis spectra of decane/sodium dodecyl benzene sulfonate (SDBS)/water emulsions were recorded for several oil phase concentrations as function of temperature. The normalized UV-Vis spectra showed that the droplet size distribution was the same for emulsions having various oil phase concentrations. However, the average diameter varies with temperature. UV-Vis absorption spectroscopy was proposed as a useful technique to analyze the behavior of chromophoric emulsifiers depending on experimental parameters such as concentration and temperature [157].

UV-Vis spectral measurements of hydrocarbons/SDBS/water emulsions in the range 300–820 nm suggested that the dimensions of the droplets were between 1 µm and 20 µm. These studies show the importance of the absorption and scattering properties obtained from the UV-Vis spectra, which give information about droplets’ shape, size distribution and chemical composition [158].

**Figure 6:**
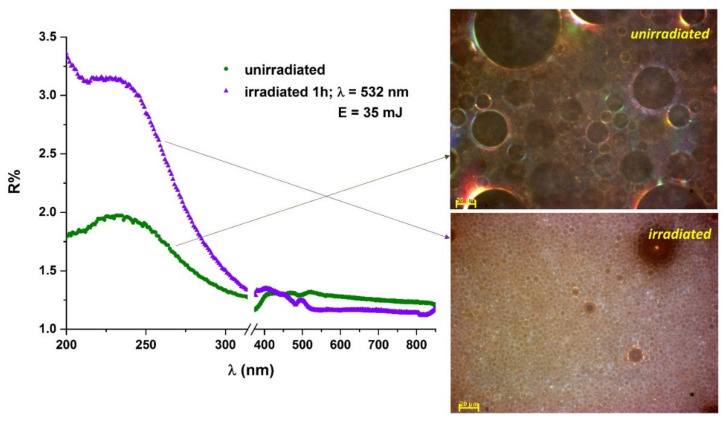
UV-Vis absorbance spectra of vitamin A and STS 10% emulsion, 1:1 ratio, before and after exposure to laser radiation. Optical microscopy images (reflected light-DIC mode, 50X magnification) of the same samples.

UV-Vis-NIR reflectance spectra of sodium tetradecyl sulfate (STS) in water and oily vitamin A emulsions showed an increase in reflectance after the sample was exposed to laser radiation, suggesting a decrease in size of the droplets (Figure 6). This finding was supported by surface tension analyses, optical spectroscopy analyses and dynamic light scattering measurements of the emulsions before and after laser irradiation [150,159].

UV-Vis absorbances recorded at two wavelengths (450 nm and 850 nm) were employed to evaluate the stability of W/O emulsions, using the turbidity ratio method. For this study, it was considered that if the components of the emulsion do not absorb at the considered wavelengths, then the turbidity (*τ*) of the emulsion is function of recorded absorbance as follows:

(1) $$\tau l=2.303\times \left(\mathrm{absorbance}\right)$$

where l represents the optical path length.

For Diesel oil emulsions, these measurements allowed to determine that the stability of the emulsion was enhanced with concentration increase in the emulsifier and that the necessaire HLB of Diesel oil is around 9 [160].

UV-Vis spectroscopy and Polarization Modulation Infrared Reflection–Absorption Spectroscopy (PM-IRRAS) helped to determine the best position of the components (Bovine Serum Albumin—BSA, Tannic Acid—TA, chitosan and pectin) in the design of a multilayer O/W emulsion. UV-Vis and PM-IRRAS spectral measurements were employed to evaluate protein/polysaccharide multilayer arrangement on a solid surface [161].

UV-Vis transmittance spectra of O/W toluene emulsions showed that their turbidity decreased over time. These measurements, completed by multiphoton ionization time-of-flight mass spectrometry (MPI-TOFMS) measurements, give information about the creaming behavior of the analyzed emulsions [162].

Other powerful tools in emulsion analysis are Fourier transform infrared (FTIR), attenuated total reflection FTIR spectroscopy (FTIR-ATR) and Raman spectroscopy. FTIR spectroscopy enables the identification of the molecular vibrations from each component of the emulsion, providing real-time information about the destabilization of the emulsion. In [159], FTIR-ATR measurements showed the influence of STS on water molecules. Even if the concentration of STS was undetectable, its action on water molecules was observed due to O–H stretching vibrations. In the same study, Raman signals revealed modifications of the C=O band at 1800 cm^−1^, allowing to observe the polymerization of STS molecules [159].

FTIR and Raman spectroscopies were also employed to study vinyl acetate-based (VAc-based) emulsions usually utilized in paintings. More exactly, FTIR-ATR and micro-Raman spectroscopy (µ-Raman) successfully identified VAc-based emulsions from different samples and determined the addition of phthalates/benzoates as plasticizers. µ-Raman measurements determined spectral markers of VAc copolymers with the monomer vinyl versatate and FTIR-ATR detected poly(vinyl alcohol) as a stabilizer of the emulsion [163].

FTIR-ATR spectroscopy was utilized to study the effect of polyglycerin-polyricinoleat emulsifier concentration on the molecular stabilization mechanisms of W/O emulsions of anthocyanin-rich bilberry extract water solution dispersed in in a medium chain triglyceride (MCT) oil phase. The modification of the O–H stretching vibration band was analyzed to evaluate the molecular interactions at water–oil interface. This study concluded that in this case, the changes in emulsifier concentration, in the range 1–10%wt, have an insignificant effect on emulsions’ stabilization [164]. For a similar emulsion, FTIR-ATR measurements showed that the emulsifier increases the intramolecular covalent O–H bonds, leading to a modification of the hydrogen bond network. This also implies a reduction of intermolecular interactions in the interfacial water layer [165].

Another study uses FTIR spectroscopy to analyze the destabilization of emulsions used in cosmetics and pharmaceutics. Reduction of the unsaturation index, increase in the carbonyl index and broadening of the C=O band are indicative of the aging of emulsions. The modification of the carbonyl band suggested that free fatty acids appear during the aging process. FTIR measurements allowed to comprehend the chemical mechanisms involved in the oxidation of these emulsions [166].

FTIR studies of sodium bis(2-ethylhexyl)sulfosuccinate (Aerosol-OT, AOT)/isooctane/water microemulsions analyzed the states of water and the conformations of AOT in these microemulsions. Four bands were recorded for O–H stretching vibrations and they were assigned to the trapped water in the palisade layer (3610 cm^−1^), the water bound to the sulfo group (3540 cm^−1^), the free water (3440 cm^−1^) and to the water bound to the sodium counterion (3225 cm^−1^). Gauche and trans conformations of AOT molecules were identified based on the absorption bands at 1739 cm^−1^ and 1725 cm^−1^, originating from carbonyl stretching vibrations [167].

O–H stretching bands were also studied to determine absorptions of bulk and interfacial water from sodium dioctyl sulfosuccinate reverse micelles. The study showed that the main absorption on the red side of the O–H band originates in the bulk water, and the interfacial water is responsible for the absorption on the blue side O–H band [168].

The modifications of O–H stretching bands were also assessed to study the structure of water in W/O microemulsions utilized to synthesize oxalate precursor NPs. NPs are obtained through a precipitation reaction in the core of the reverse micelles formed when two initial microemulsions are mixed. In order to identify the water structure, the O–H stretching band was decomposed into three components, each corresponding to a different type of hydrogen bonding. The findings lead to the conclusion that after the synthesis of NPs, the number of bound water molecules was increased [169].

FTIR spectroscopy was one of the techniques employed to determine the structural changes of proteins incorporated in W/O emulsions. This method allowed to determine that the secondary structures of BSA and human serum albumin (HSA) changed after their incorporation in emulsions [170]. ATR-FTIR measurements allowed to determine the heat-induced modification in the structure of edible coconut protein concentrate (CPC), that is also used as oil-in-water emulsifier [171].

The effect of temperature on emulsion stabilized by soy lecithin was studied also through FTIR spectroscopy. Analysis of bands originating in –OH vibration, –CH_2_ stretching, H–O–H bending vibrations, and P=O, C–O–C, and P–O–C vibrations allowed to determine that the emulsions stabilized by phospholipids remained stable when the temperature was varied, as opposed to the control emulsion that had no emulsifier added [172].

FTIR spectroscopy was useful in determining the chemical groups in the crude oils responsible for emulsifications. This study is important for separation of oil from O/W emulsions, which is a significant problem for the petroleum industry [173].

As in the case of foams, the internal dynamics and structure of emulsions can be studied with spectroscopy techniques based on multiple scattering of light, like DWS.

Marze et al. employed DWS in back- and forward-multiple scattering to evaluate the in vitro digestion of eight emulsion samples, determining that the type of triglyceride in the emulsions is the main parameter to influence the digestion. The advantage of using DWS is that the emulsions can be analyzed at their normal appearance, without the need to dilute them. When comparing the particle size distribution (PSD) determined through DLS with the PDS determined from DWS measurements for multiple scattering, Marze et al. found the results to be in good agreement. In order to determine the PDS, the statistical analysis of cumulants and moments employed for single scattering was applied to DWS data. This method could not have been successfully applied to long term digestion. Forward-scattering DWS measurements, complementary to nuclear magnetic resonance diffusion measurements, permitted to determine the diffusion coefficients. It was observed that during digestion, the transitions were from a droplet to a vesicle and afterwards to a micelle [174].

DWS has the potential to monitor the manufacturing process of turbid pharmaceutical emulsions, being able to offer information about the dynamics and the statics of the emulsions. Continuous DWS analysis during generation of pharmaceutical emulsions can give data about optimal homogenization conditions, showing when to stop the manufacturing process in order to prevent overprocessing of emulsions. Emulsion dynamics correlated with static analysis were in agreement with the modification of the droplet size distribution, during emulsion generation [175].

A series of model pharmaceutical emulsions were analyzed through DWS and the results were compared to other stability analysis methods. Obtained results regarding the stability were similar to those from the other methods. This, along with the fact that the technique is non-invasive, fast and needs only small volumes of emulsions, makes DWS suitable for analyzing the stability of pharmaceutical emulsions [176].

A new model for fitting DWS measurements of emulsions during their creaming/sedimentation is presented in [177]. This model starts from a Monte Carlo simulation of the light that diffuses in the volume of the emulsion in order to determine the averages and the distributions of the droplet size and dynamics.

DWS proved to be a useful technique not only in pharmaceutics, but also in cosmetics. The stability of cosmetic formulations was assessed via DWS and it was observed that the instability of the emulsion was higher for larger values of mean square displacement (MSD) [178].

The studies selected for this review demonstrate that the presented spectroscopy techniques are powerful tools in investigating emulsions and have applicability in numerous fields like cosmetics, pharmaceutics, medicine, food industry, fuel and oil enhanced recovery, etc.

## 4. Spectral Characterization Techniques

Bubbles/droplets size and distribution, which are connected with foam/emulsion characteristics, are influenced by the nature of the components and their physicochemical features. In this respect, spectral analysis brings important information about their structures, chemical stability, time stability, and molecular data. Some of the techniques for spectral characterization of foams and emulsions are discussed in this chapter.

### 4.1. Optical Absorption Spectroscopy

The ability to absorb, transmit or reflect light are among the most important aspects in determining the characteristics of sample constituents, which have a direct impact on foam and emulsion properties. As a result, researchers typically analyze the absorbance and reflectance spectra of emulsions and correlate the findings to the perceptible appearance of the system. [158,179,180].

#### 4.1.1. Absorption Spectroscopy

As a rule, the type of ion or molecule being examined determines the wavelength at which a chemical component absorbs light. Through the Beer–Lambert equation, the amount of light absorbed is proportional to the chemical’s type and concentration [181].

The optical absorption spectroscopy uses electromagnetic radiation specific to UV (190–400 nm) and Vis (400–800 nm) spectral ranges. It is also known as electronic spectroscopy because the absorption of UV-Vis radiation by a molecule causes a change among the molecule’s electronic energy states.

The origin of these absorptions are the valence electrons, and they can be generally found in one of three types of electron orbitals: (*) single (*σ* bonding orbitals); (**) double or triple bonds (*π* bonding orbitals); and (***) nonbonding orbitals (*n*), (lone pair electrons). The *σ* bonding orbitals tend to be lower in energy than the *π* bonding orbitals, which in turn show lower energy than nonbonding orbitals. When electromagnetic radiation of the correct frequency is absorbed, a transition occurs from one of these orbitals to an empty orbital, usually an antibonding orbital, σ* or π*. The exact energy differences between the orbitals depend on the atoms present and the nature of the bonding system, thus most of the absorptions observed involve only *π*→π*, n→σ*, and *n*→π* transitions [182].

The experimental technique is based on the detection of the absorbance and the turbidity level of colloidal samples using a spectrophotometer. Spectrophotometry allows to determine how much light is absorbed by a substance through measurement of the intensity of light as it travels through a solution sample. Most of the commercial spectrophotometers use one of three basic designs: a fixed spectrophotometer equipped with a single sample holder and light beam, a scanning spectrophotometer with dual sample holders and light beams for simultaneous analysis or a non-scanning spectrophotometer with the capacity to measure multiple wavelengths [183]. The instrumentations are made of up to five components: (1) a stable source of radiant energy; (2) a wavelength selector that isolates a limited region of the spectrum for measurement; (3) one or more sample containers; (4) a radiation detector, which converts radiant energy to a measurable electrical signal; and (5) a signal processing and readout unit, usually consisting of electronic hardware and a computer.

To conduct analysis, a sample is placed in a quartz cuvette, where the electromagnetic wave at the selected spectral range would perform a thorough scanning of the emulsion sample. Once the scanning is complete, the absorbance/transmittance spectra of measured values are corrected using the value for the reference sample (the dispersive environment), usually deionized or distilled water [150,179].

#### 4.1.2. Reflectance Spectroscopy

Reflection of electromagnetic radiation is the change in direction of a wavefront at an interface between two different media so that the wavefront returns into the medium from which it originated. When light is reflected, its properties and characteristics may differ. The strength of the light’s field, its wavelength, and the sample itself all contribute on how light is influenced by matter. The interaction of light with matter is also controlled by other factors, such as temperature, pressure and other external fields (electrical, magnetic).

Uneven surfaces, such as foams and emulsions, reflect light back in all directions. Thanks to the diffuse character of the reflection, the illuminated object might be seen from every angle. Diffuse reflection occurs when an incident ray of light strikes a surface, and the light is scattered. In an ideal diffuse reflection, all the light is perfectly distributed in a hemisphere of even illumination around the point where the light hits the diffusion surface. The most often used theory to describe and analyze diffuse reflectance spectra is the Kubelka–Munk theory [184].

Most diffuse reflectance spectra collected in UV-Vis-NIR spectral ranges are performed using an integrating sphere as an optical module of a spectrophotometer. The integrating sphere is a hollow enclosure with walls constructed of a diffusely reflecting material that reflects all wavelengths of interest with a high reflecting power. The modern integrating spheres come with their own durable coatings already applied.

As most of the UV-Vis-NIR spectrophotometers are now double-beam, dual-beam, or diode array instruments, the integrating spheres are designed accordingly. In an integrating sphere accessory for a double-beam spectrophotometer the chopped signal from the source is separated into sample and reference beams. The sample and reference are measured concurrently, thus allowing for a comparison measurement. An integrating sphere accessory for a dual beam spectrophotometer requires a substitution measurement. The mechanical chopper is replaced by a beam splitter. Half the beam is sent toward the original instrument detector, while the other half is sent to the integrating sphere set up for a substitution measurement. Diode array spectrometers have gradually become more popular because of the increased measurement speed. A reflectance spectrum using a diode array spectrometer contains the source inside the sphere to produce polychromatic illumination. Collection of a diffuse reflectance spectrum using a diode array instrument requires a substitution measurement, as in the case of the dual-beam instrument.

Laser methods are unsuitable for reflectance measurements of foams because they are too localized, and the integrating sphere needs modification for liquids but the 0°/45° arrangement is an ideal and accepted geometry for diffuse reflectance measurement [185]. In this respect, Aziz et al. explored the long-term stabilization of reflective foams in sea water using such an arrangement. The study aimed at using the oceanic foams to increase planetary albedo considering that extending foam lifetime moderates the energy required to maintain large areas of ‘ocean mirror’ [186].

### 4.2. Vibrational Spectroscopy

Vibrational spectroscopy, known also as molecular spectroscopy is an important analytical tool to investigate foams and emulsions properties. Two types of vibrational spectra are mostly used in the analysis of colloids, namely infrared (IR) and Raman spectra, which are generated by different types of energy exchanges between the molecules under study and electromagnetic radiation. In IR spectroscopy, a photon is absorbed as a result of a vibrational transition involving a change in dipole moment. The difference in energy between the two vibrational states of the molecule is equal to the energy of the absorbed photon.

The electromagnetic field induces a dipole moment in the molecule in Raman spectroscopy, resulting in an energy exchange that occurs concurrently with the vibrational transition. Because the energy of the excited photons is higher than the energy difference between the two vibrational states, the interaction with the field produces a scattered photon with a frequency shift equal to the energy difference between the vibrational states [187,188].

The vibrational spectroscopy generates specimen-specific chemical information. Although IR absorption and Raman scattering spectroscopy provide complementary information about molecular vibrations, they are significantly different techniques and require very different instrumentation to measure their spectra.

#### 4.2.1. Infrared Spectroscopy

Infrared spectroscopy is based on the absorption of radiation in the IR spectrum region by vibrations between atoms in molecules, and hence offers information about the chemical composition and conformational structure of the samples under investigation. This investigation technique allows qualitative (structural elucidation and compound identification), as well as quantitative (based on the Lambert–Beer law, according to which the intensity of absorption bands is proportional to the concentration of each component in a homogeneous mixture) interpretation of the results [189]. The standard IR spectrometer broad band source emits all of the IR frequencies of interest at the same time, with the near-IR area ranging from 14000 to 4000 cm^−1^, the mid-IR region from 4000 to 400 cm^−1^ and the far-IR region ranging from 400 to 10 cm^−1^. The IR spectrum is generated as a plot of the absorbance/transmittance intensity against the wavenumber, which is proportional to the energy difference between the ground and excited vibrational states [190].

In order to address the constraints of IR dispersive equipment, Fourier transform infrared (FTIR) spectroscopy was developed. The key challenge was the slower scanning procedure, which was overcome with the help of the interferometer. The observed interferogram signal cannot be analyzed directly, thus the well-known Fourier transform mathematical technique is applied by a computer to obtain the desired spectral information for analysis.

A FTIR spectrometer usually consists of four major elements: the radiation source (specific to different spectral ranges), the interferometer, the sample compartment, and the detector, which are specially designed to measure specific interferogram signal. Additionally, reflection gratings, and mirrors are used in FTIR instruments.

A background spectrum must be assessed in order to establish a relative scale for absorption intensity. Normally, no sample is in the beam when this measurement is made. To determine the percent transmittance, the background is compared to the measurement taken with the sample in the beam.

The use of attenuated total reflectance (ATR) in FTIR spectroscopy has become the primary sampling method for FTIR spectroscopy. The major advantage is the lack of sample preparation for samples. When light reflects off certain materials (diamond, ZnSe, etc.) at a critical angle, the light undergoes total reflectance with a small amount of light being absorbed into the sample in contact with the crystal surface. The penetration depth depends on the refractive index of both the sample and the crystal itself. Since the refractive index is dependent on wavelength, spectra taken with ATR have slightly different intensity ratios across the spectrum and may need to be corrected to compare to transmission spectra [191,192].

#### 4.2.2. Raman Spectroscopy

Light scattering phenomena may be classically described in terms of electromagnetic radiation produced by oscillating dipoles induced in the molecule by the electromagnetic fields of the incident radiation. When a beam of monochromatic light interacts with a molecule, a portion of the radiation energy is scattered. A small amount of this scattered light, approximately 1 x 10^−7^, called Raman scattering, presents an inelastic character and has a frequency (*hν_o_* + *hν,* anti-Stokes lines or *hν_o_* − *hν,* Stokes lines) different from the frequency of the incident radiation (*hν* is the energy difference between vibrational states) [193].

Raman spectroscopy is a versatile technique, which makes use of scattered light to identify molecular vibrations. It can provide details about a molecule’s structure, symmetry, electronic environment and bonding, allowing for quantitative and qualitative evaluation of specific compounds. Raman spectra results as emission excited by monochromatic radiation in UV (50000–25000 cm^−1^), Vis (25000–14300 cm^−1^), or NIR (14300–4000 cm^−1^) spectral ranges. They are due to modulation of incident light by molecular vibrations [188].

Dispersive single-stage spectrographs (axial transmissive or Czerny–Turner monochromators) paired with CCD detectors are most common although Fourier transform spectrometers are also common for use with near-IR lasers. Generally, the experimental arrangement for Raman spectroscopy consists of four major components: the excitation source, the sample illumination system and light collection optics, the wavelength selector (gratings and filters) and the detector.

Solid state lasers are widely employed in current Raman equipment, emitting radiation with wavelengths of 532 nm, 785 nm, 830 nm and 1064 nm. The shorter wavelength lasers have higher Raman scattering cross-sections, so the resulting signal is greater. However, drawbacks associated with the incidence of fluorescence appear, also in situation of shorter wavelengths [183]. The fluorescence background in Raman spectroscopy can be effectively suppressed by using pulsed lasers and time-gated detectors. A recent solution to reduce the high complexity and bulkiness of the time-gated systems is to implement the detector by utilizing time-resolved single-photon avalanche diodes (SPADs) fabricated in complementary-metal-oxide-semiconductor (CMOS) technology [194].

The main challenge of Raman spectroscopy is discriminating it from the intense Rayleigh scattering. To achieve this, optical filters are used to prevent unwanted light from reaching the spectrometer while allowing the relatively weak Raman signal to pass through.

### 4.3. Diffusing Wave Spectroscopy

DWS is a multiple light scattering technique that has gained considerable attention since its introduction in the late 1980s [195] as a fast, non-destructive and reproducible means to measure the structure, dynamic, and viscoelastic properties of highly turbid environments like foams and emulsions [114].

The assumptions and approximations in the DWS theories are covered in detail in the literature [196,197,198]. The transport of light is modeled as a random walk having an optical transport mean free path l*. The diffusion equation is then applied to a specific sample-cell geometry, which typically has a thickness far in excess of l*, while considering the illumination and detection configuration used [199].

The intensity of the unscattered portion of the incident beam decays exponentially with distance into the medium according to the scattering mean free path *l_s_,* whose value is set by both the number density (ρ) and total scattering cross section (σ) of the suspended particles *l_s_* = 1/ρσ in the standard DWS theory. In the multiple scattering regime, the sample is very large comparing with *l_s_*, so that photons scattered away from the incident beam typically experience many more scattering events before exit the sample. The transport mean free path l*, which enters into diffusion theory treatment of this process, is related to the scattering mean free path according to how strongly photons are deflected from their unscattered, or forward, direction: l* = l_s_/1−cosθ, where *ϴ* is the deflection angle and the average is taken over the scattering form factor for the probability of scattering by *ϴ* [200].

Even while most experimental samples have a white appearance and do not allow photons to pass through without scattering several times, the scattering signal is weak in the sense that subsequent events are uncorrelated and the contributions from photons crossing distinct paths increase incoherently. This statement simplifies the theories of DWS [195,201], although they are still highly complex and involve several uncontrolled approximations and adjustable parameters. In [200] the accuracy of DWS predictions for the normalized electric field autocorrelation function by means of computer simulation, and guidelines for the analysis of transmission experiments were done. Results are obtained for the accuracy of both characteristic decay scale and functional form of function *g_l_(τ*) for a variety of experimental situations, which are both on the order of a few percent. The details of reported results are important for the experimentalists wishing to know and to minimize the systematic error introduced during DWS data analysis.

Motivated by the continuous improvement of DWS techniques [113,202,203] and the need to rectify collective scattering effects in DWS of dense colloidal systems, which can adversely affect passive microrheology, a systematic experimental comparison of the plateau elastic shear moduli measured using both mechanical rheometry and modern DWS microrheology of disordered, microscale monodisperse emulsions, as a function of droplet volume fraction was reported [204]. In addition, DWS in a standard Dynamic Light Scattering setup was published [205].

## 5. Conclusions

The optical properties of foams and emulsions are of interest both for basic research and for advanced applications in biomedicine, life sciences, food science, unconventional technologies in energy production and raw materials extraction, to mention a few of their applications. Based on literature reports, the spectral measurement of the fine molecular structure of foam and emulsion constituents can reveal essential information about the mechanisms involved in the formation of bubbles and droplets, as well as their behavior in relation to many external conditions that can influence the final features. This is based on the fact that, in general, upon interaction with optical beams in the visible and NIR spectral ranges, the obtained information is collected mainly from the beam focus within the sample. This may be made using long or short optical paths in the specimens, i.e., longer waist or shorter waist samples. A typical example is constituted by the use of spectrophotometric cells that have thicknesses between 1 mm to 10 mm or more and on which one may send either plane parallel or focused beams.

A longer optical path in an emulsion or foam sample is obtained by the reflection and scattering of the optical beam within it. This would produce a more intense optical signal from such a sample than from a liquid sample, for instance, if comparable dimensions of samples are considered. According to the Lambert–Beer law, the larger the optical path the more important absorption signal may be measured. As a result, if the sample is a foam/emulsion, the absorption signal is increased when compared to the signal measured on the same sample as liquid.

The FTIR investigations of foams and emulsions enable the identification of absorption bands unique to each of the components, as well as a real-time assessment of their stability.

Raman spectroscopy allows the characterization of the modifications at the molecular level, through identification of the corresponding vibrations from the functional groups of the foam or emulsion’s components. The Raman intensity of vibrations associated with particular bonds in molecular compounds is very high, and bands resulting from these modes contain the majority of structural information. Laser sources have also advantages arising from their high fluence and characteristic propagation properties. These properties produce radiation capable of being focused on very small volumes (having even micrometers scales), that permits sample characterization with accuracy, speed and high spatial resolution. Raman spectroscopy can easily access vibrations originating from skeletal deformation modes or interchain interactions, which can reveal important data about the 3D microscopic structures like bubbles, intern micro-/nanodroplets, nanoparticles. In this way, emulsions and foams provide highly sensitive bases to measure, for instance, low concentrations of molecules down to the level of traces and open new perspectives in microfluidics.

DWS, as a modern optical technique derived from dynamic light scattering, allows the measurements of the complex phenomena in strongly scattering media like foams and emulsions. One of the most significant advantages of this technique is that it enables measurement of the dynamics over a large range of time scales (10^−7^ to 10 s) and at short length scales (down to ~1 nm). Moreover, DWS can be used to perform non-invasive microrheology, extracting rheological properties on the micron scale without making actual contact with the sample.

As processing power and analysis capabilities have improved, the utility of spectroscopic techniques has expanded significantly and thus effectively complements the other characterization techniques of foams and emulsions.
